# Jefferson Medical College

**Published:** 1860-03

**Authors:** 


JEFFERSON MEDICAL COLLEGE.—The catalogue of this institution, just issued, presents the names of six hundred and thirty students, constituting the largest class ever assembled within her walls.

